# Juice of *Citrullus lanatus var. citroides* (wild watermelon) inhibits the entry and propagation of influenza viruses in vitro and in vivo

**DOI:** 10.1002/fsn3.2023

**Published:** 2020-11-27

**Authors:** Ryosuke Morimoto, Kae Yoshioka, Miyu Nakayama, Emiko Nagai, Yoshinobu Okuno, Ayaka Nakashima, Taro Ogawa, Kengo Suzuki, Toshiki Enomoto, Yuji Isegawa

**Affiliations:** ^1^ Department of Food Sciences and Nutrition School of Human Environmental Sciences Mukogawa Women’s University Nishinomiya Japan; ^2^ Faculty of Human Life Science Shikoku University Tokushima Tokushima Japan; ^3^ Department of Food Science Ishikawa Prefectural University Nonoichi Japan; ^4^ Osaka Institute of Public Health Osaka Japan; ^5^ Euglena, Co., Ltd Minato‐ku Japan; ^6^ Present address: Faculty of Human Life Science Shikoku University Tokushima Tokushima Japan

**Keywords:** influenza virus, virus infection inhibition, wild watermelon

## Abstract

Vaccines and various anti‐influenza drugs are clinically used to prevent and treat influenza infections. However, with the antigenic mismatch of vaccines and the emergence of drug‐resistant viral strains, new approaches for treating influenza are warranted. This study focused on natural foods as potential candidates for the development of new treatment options for influenza infections. The screening of plants from the Cucurbitaceae family revealed that the juice of *Citrullus lanatus var. citroides* (wild watermelon) had the strongest ability to inhibit the replication of influenza virus in Madin–Darby canine kidney cells. The results of a time‐of‐addition assay indicated that wild watermelon juice (WWMJ) inhibits the adsorption and late stages of viral replication, suggesting that WWMJ contains multiple constituents with effective anti‐influenza activity. A viral adsorption analysis showed that WWMJ reduces the amount of viral RNA in the cells at 37°C but not at 4°C, confirming that WWMJ inhibits viral entry into the host cells at 37°C. These results suggest that a mechanism other than the inhibition of viral attachment is involved in the anti‐influenza action of WWMJ, which is perhaps responsible for a reduction in internalization of the virus. Administration of WWMJ into the nasal mucosa of BALB/c mice infected with the A/PR/8/34 mouse‐adapted influenza virus was seen to significantly improve the survival rate. The findings of this study, therefore, demonstrate the anti‐influenza potential of WWMJ in vitro and in vivo, thereby suggesting the candidature of WWMJ as a functional food product that can be used to develop anti‐influenza agents and drugs.

## Graphical abstract

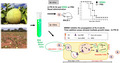

## 1 INTRODUCTION

Influenza virus infections spread at epidemic levels worldwide every year, and sometimes become pandemics. In 2009, a strain of the influenza A virus (H1N1) rapidly spread worldwide, and the global pandemic alert level was raised to phase 5, indicating the sustained human‐to‐human transmission of a novel influenza strain of animal origin (Fraser, 2009).

Influenza viruses are RNA viruses of the family *Orthomyxoviridae*. Type A, B, and C influenza viruses can infect humans, among which types A and B cause seasonal epidemics. Type A influenza viruses have eight single‐stranded RNAs that encode 13 proteins (Jagger, 2012), and are classified into subtypes based on the antigenicity of the hemagglutinin (HA) and neuraminidase proteins on the viral membrane (Webster, 1992). Type A influenza causes epidemics every year, resulting in severe respiratory disease symptoms, such as sneezing, sore throat, fever, headache, muscle fatigue, and malaise in infected individuals (García, 2006).

The declining birth rate and aging population of Japan has caused influenza virus infection to be recognized as an increasingly serious health risk. The appearance of resistant viruses and the infection of humans with avian influenza viruses are also considered as noteworthy risks (Dortmans, 2013). In recent years, the emergence of new viruses has been reported; type D Influenza virus, for example, infects not only bovine and porcine bronchial epithelial cells, but also human bronchial epithelial cells, and therefore has the possibility of zoonotic infection (Song, 2016).

Several drugs are used to treat influenza virus infections, including M2 channel blockers (amantadine and rimantadine) and neuraminidase inhibitors (zanamivir and oseltamivir). These drugs inhibit viral growth after the virus has been adsorbed into the host cells (and viral RNA has been released into the cell) and therefore restricts the release of viruses into the extracellular space. However, recent reports have indicated that some influenza A viruses are resistant to M2 channel blockers (Belshe, 1989) and neuraminidase inhibitors (Bloom, 2010; Le, 2005; Seibert, 2012); therefore, new prevention and treatment regimens for influenza virus infections are required.

Recently, a new anti‐influenza drug, favipiravir, an RNA polymerase inhibitor, was developed as a treatment for influenza infections (Furuta, 2002; Kiso, 2010; Vanderlinden, 2016). Further, baloxavir marboxil, an endonuclease inhibitor, is being developed (O'Hanlon, 2019). Over the past several years, the anti‐influenza effects of food extracts have been reported, which also have anti‐inflammatory (Danciu, 2018) and antioxidant (Ehala, 2005; Kamei, 2016; Lee, 2012; Park, 2013; Sekizawa, 2013) activities. Based on such previous findings, the present study focused on the anti‐influenza effects of functional foods, such as wild watermelon juice. It has been previously reported that adlay tea is effective against influenza virus (Nagai, 2018), and thus we continued to search for more food extracts that are effective against this virus in the present study (Horio, 2020; Nagai, 2019).

Wild watermelon (WWM), *Citrullus lanatus var. citroides*, which is native to the Kalahari desert in southern Africa, can adapt to and grow under severely dry and high‐ultraviolet‐light conditions. WWM is used as a dietary source of hydrogen and water in its native region, and its seeds are also known to contain many essential amino acids (Umar, 2013). WWM has a high citrulline content, which protects the plant from the stresses of its native environment (Akashi, 2004; Takahara, 2005; Yokota, 2002). Although there have been several reports of the benefits of WWM, its food functionality is still a relatively new field of research.

In the present study, Madin–Darby canine kidney (MDCK) cells and BALB/c mice infected with the A/PR/8/34 mouse‐adapted influenza virus were used to demonstrate the in vitro and in vivo anti‐influenza activity of WWM juice (WWMJ). Time‐of‐addition assay and viral adsorption analysis were used to investigate the inhibition of viral replication and to assess the amount of viral RNA in cells under different temperature conditions, respectively. The focus‐forming reduction assay and cell viability tests enabled the determination of viral activity. The findings of this study have emphasized the potential candidature of natural foods, such as WWMJ, as alternate therapeutic options for severe viral infections, such as that caused by the influenza virus.

## 2 MATERIALS AND METHODS

### 2.1 Cell lines and viruses

Madin–Darby canine kidney cells were grown in Eagle's minimum essential medium (MEM; Wako Pure Chemical Industries, Ltd) containing 7% fetal bovine serum (FBS). Monkey kidney (CV‐1) cells were cultured in Dulbecco's modified Eagle's medium (DMEM; Wako) containing 10% FBS. Type A influenza viruses H1N1 (A/Puerto Rico/8/34, A/New Caledonia/20/99, A/Beijing/262/95, A/Suita/6/2007, A/Suita/114/2011, A/Osaka/2024/2009, A/Osaka/71/2011) and H3N2 (A/Sydney/5/97, A/Suita/120/2011) and type B influenza viruses (B/Nagasaki/1/87, B/Shanghai/261/2002) were used in the experiments. The virus culture was diluted in serum‐free MEM containing 0.04% bovine serum albumin (BSA, fraction V; Sigma‐Aldrich) and then incubated with the cells to infect them at a multiplicity of infection (MOI) of 0.001 for 1 hr at 37°C. The medium was then removed and replaced with serum‐free DMEM containing 0.4% BSA and 2 µg/ml acetyl trypsin (Merck Sigma‐Aldrich) for the rest of the infection period.

### 2.2 Preparation of watermelon extracts and other sample extracts

Wild watermelon juice and the juice from commercially available watermelon (WMJ) were tested for their anti‐influenza activity in this study. WWMJ was provided by Euglena, Co., Ltd. WWMJ was treated at 80°C for 30 min, and the proteins were removed to establish if lectins participated in the antiviral activity. WMJ was squeezed from watermelons obtained from a Hitorijime cultivar produced in Kumamoto and purchased from a supermarket. WWMJ and WMJ were centrifuged at 1,600 ×g and the supernatants freeze‐dried. The dry weight of each resulting powder was measured and then dissolved in 20 mg/ml of ultrapure water. The samples were then sterilized by filtration through a Millex GX membrane with a 24 mm diameter and a pore size of 0.45 µm (Merck Millipore) and stored at − 30°C until analysis. Other sample foods of Cucurbitaceae, winter squash (*Cucurbita maxima*) and zucchini (*Cucurbita pepo* L.), were purchased from a supermarket. The samples were cut and freeze‐dried. Next, the dried samples (5 g) were powdered, mixed with water (50 ml), and extracted in a hot water bath for 60 min at 80°C. Subsequent steps after the centrifugation were the same as described above.

### 2.3 Viral yield determination in the presence of watermelon samples

The effects of the addition of food samples on viral yield were determined using a modified version of the previously described procedure (Nagai, 2018). MDCK cells were cultured in a 24‐well plate (Thermo Fisher Scientific) at 1 × 10^5^ cells/well in 500 µl/well MEM containing 7% FBS and incubated for 24 hr at 37°C. The confluent monolayers of cells were then rinsed twice with serum‐free MEM. Diluted virus culture was incubated with the cells at a MOI of 0.001 for 1 hr at 37°C. The infected cells were rinsed once with serum‐free MEM after 1 hr and then cultured in DMEM containing watermelon extract (500 µl/well). Supernatants were collected after 24 hr as the influenza virus samples and used in a focus‐forming assay.

### 2.4 Focus‐forming reduction assay (FFRA) of viral activity

The FFRA was performed according to a slightly modified version of the previously described method (Nagai, 2018 and Okuno, 1990). MDCK cells (approximately 10^4^ cells/well) were seeded in 96‐well flat‐bottom plates (Corning Inc.) and incubated in 5% CO_2_ at 37°C to form monolayers. The viral solutions were serially diluted 10‐fold in 96‐well round‐bottom plates (Thermo Fisher Scientific) in MEM containing 0.04% BSA on the following day. MDCK cells in 96‐well flat‐bottom plates were washed twice with serum‐free MEM and then 30 µl of each viral mixture was added to each well. Samples were incubated for 1 hr at 37°C. The viral solutions were then removed and cells were washed with serum‐free MEM, covered with 100 µl of MEM mixture (equal volumes of FBS‐free MEM and Avicel^Ⓡ^ RC‐591 NF; FMC Health & Nutrition) containing 0.4% BSA, and incubated for 18 hr at 37°C. The supernatant was then removed and cells were washed with serum‐free MEM and fixed with absolute ethanol at room temperature for 10 min. The ethanol was then removed completely. Cells that were not stained immediately were stored at −80°C until staining.

Focus staining was performed by adding 50 µl of murine monoclonal anti‐HA antibody [ C179 for A (H1N1) viruses, Okuno, 1993; F49 for A (H3N2) viruses, Ueda et al., 1998; and 7B11 for B viruses, Nakagawa, 1999 ] and a goat antimouse IgG antibody conjugated to horseradish peroxidase (Merck KGaA). The peroxidase reaction was developed for 30 min according to the procedure given by Graham and Karnovsky (1966), using 0.1% H_2_O_2_ and 0.3 mg/ml 3,3′‐diaminobenzidine tetrahydrochloride (Wako) in phosphate‐buffered saline (PBS). Cells were rinsed with water and dried with a hair dryer after the reaction. The numbers of foci in immunostained infected cells were determined under an inverted light microscope.

### 2.5 Time‐of‐addition assay

A time‐of‐addition assay was performed using a modified version of the previously described procedure (Nagai, 2018). MDCK cells were plated in 24‐well plates as described above, rinsed twice with serum‐free MEM, and then inoculated with A/PR/8/34 (MOI = 0.01). Cells were rinsed twice with serum‐free MEM and incubated in DMEM after 1 hr as described above. DMEM containing 1 mg/ml WWMJ, which is approximately ten times the median inhibitory concentration (IC_50_) (Table 1), was added at different time points: within the 12 hr period prior to infection (‐12–‐1 hr, pretreatment), between 1 hr prior to infection and time of infection (‐1–0 hr, adsorption), and between 0–2 hr, 2–4 hr, 4–6 hr, 6–8 hr, or 0–8 hr after infection (replication), as shown in Figure 2a. Cell monolayers were rinsed twice with serum‐free MEM after each incubation period and the medium was replaced with fresh medium. Cells were cultured for 8 hr after infection. Infected cells were then frozen at −80°C and subjected to two freeze–thaw cycles before the viral yield was determined with the focus‐forming assay.

**TABLE 1:** Effect of WWMJ on the multiplication of various influenza virus types and strains

Virus type and strain	IC_50_ (mg/ml or ng/ml)^a^		CC_50_ (mg/ml or µg/ml)^b^		SI^c^	
	WWMJ	Oseltamivir acid	WWMJ	Oseltamivir acid	WWMJ	Oseltamivir acid
A (H1N1)						
PR/8/34	0.10 ± 0.05	0.45 ± 0.01	>10	>200	>100	>444,444
New Caledonia/20/99	0.20 ± 0.03	0.38 ± 0.18	>10	>200	>50	>526,315
Beijing/262/95	0.11 ± 0.03	ND	>10	ND	>90	ND
Suita/6/2007	0.14 ± 0.03	ND	>10	ND	>71	ND
Suita/114/2011	0.19 ± 0.08	ND	>10	ND	>52	ND
Osaka/2024/2009^d^	0.13 ± 0.01	180 ± 59	>10	>200	>76	>1,111
Osaka/71/2011^d^	0.20 ± 0.03	259 ± 75	>10	>200	>50	>772
A (H3N2)						
Sydney/5/97	0.87 ± 0.07	ND	>10	ND	>11	ND
Suita/120/2011	0.71 ± 0.02	ND	>10	ND	>14	ND
B						
Nagasaki/1/87	0.88 ± 0.25	6.10 ± 1.19	>10	>200	>11	>32,786
Shanghai/261/2002	0.41 ± 0.06	ND	>10	ND	>24	ND

Note

ND is not determined.

^a^ Values are the average of results obtained using various final concentrations of WWMJ and MDCK cells, from three independent experiments. The IC_50_ of WWMJ and that of oseltamivir acid (Nagai, 2018) are given in mg/ml and ng/ml, respectively.

^b^ Values are the average of results obtained using various final concentrations of WWMJ and MDCK cells, from three independent experiments. The CC_50_ of WWMJ and that of oseltamivir acid (Nagai, 2018) are given in mg/ml and µg/ml, respectively.

^c^ Selectivity index = CC_50_/IC_50_.

^d^ Oseltamivir‐resistant virus.

### 2.6 Cell viability determination

Cell viability was determined with a Cell Proliferation Kit I (MTT) (F. Hoffmann–La Roche Ltd). The cytopathic effects in the virus‐infected cells to which various concentrations of WWMJ had been added were observed under a microscope.

### 2.7 Hemagglutination inhibition (HI) test

The HI test was conducted using receptor‐destroying enzyme‐treated guinea‐pig red blood cells in 96‐well U‐bottom plates (Thermo Fisher Scientific) with the standard microtiter assay as described previously (Dowdle, 1979).

### 2.8 Viral adsorption inhibition assay

The amount of virus attached to the cells was determined by measuring the viral RNA encoding the M protein (MP). Viral RNA bound to cells was extracted, and cDNA synthesized and viral RNA quantified as described previously (Nagai, 2018).

### 2.9 Cell fusion inhibition test

Cell–cell fusion was analyzed using the method previously described (Nagai, 2018). Briefly, the influenza virus solution (A/PR/8/34: MOI of 0.001) was added to CV‐1 cells and cultured for 24 hr. Infected cells were washed twice and incubated for 15 min in DMEM containing 10 µg/ml trypsin. Cells were washed twice and incubated for 30 min in DMEM containing 1 mg/ml WWMJ. Next, cells were washed and treated with fusion medium to adjust the pH to 5.0 and incubated for 2 min. Thereafter, cells were washed twice and incubated for 3 hr with DMEM containing 2% FBS. Cells were then stained with Giemsa and the number of fused cells counted.

### 2.10 Mice and infection experiment

Five‐week‐old female BALB/c mice (20–22 g) were obtained from Charles River Laboratories International, Inc. Mice were maintained in a laminar flow hood at the Animal Experiment Facility of the Hyogo College of Medicine, Hyogo, Japan, under suitable conditions (12‐hr light/dark cycle, temperature 25°C, relative humidity 50%) and fed AIN‐76 purified mouse diet (CLEA Japan, Inc.) for 1 week. Mice were divided into two groups (test group and control group) containing 10 mice each. One day prior to infection, 20 μl of WWMJ (20 mg/kg/day) was inoculated into one nostril of the 6‐week‐old mice in the test group; the control group was given an equal volume of PBS. On the following day, mice were infected with 100 FFU/mouse of A/PR/8/34 influenza virus, a mouse‐adapted strain, by inoculating 20 μl of the virus solution into one nostril. WWMJ (20 µl) was inoculated nasally into the sample group and 20 µl of PBS into the control group after 1 hr of virus inoculation. WWMJ and PBS treatments were repeated daily for the next 3 days. Body weights and survival rates of the WWMJ‐ and PBS‐treated mice were determined. Survival was monitored for 14 days after infection. Animal husbandry was performed in accordance with the ordinance of the Regulation for Enforcement of the Act on Welfare and Management of Animals relating to the care and management of experimental animals (Ordinance, 2016).

### 2.11 Statistical analyses

Statistical analyses were performed using unpaired *t*‐test and analysis of variance with Tamhane test using the SPSS version 24.0 (SPSS, Inc.). The amounts of virus determined in the time‐of‐addition assay and the antiviral assay were analyzed by the Student's *t*‐test in Excel Toukei version 6.0 (Esumi). The survival rates of the mock‐ and virus‐infected mice were analyzed by the Kaplan–Meier method and log‐rank test in Excel Toukei Statcel 3 (OMS). Values are presented as means ± standard deviations (*SD*). A *p*‐value (*p*) < .05 was considered statistically significant.

## 3 RESULTS

### 3.1 WWMJ inhibits influenza viral growth

**FIGURE 1:**
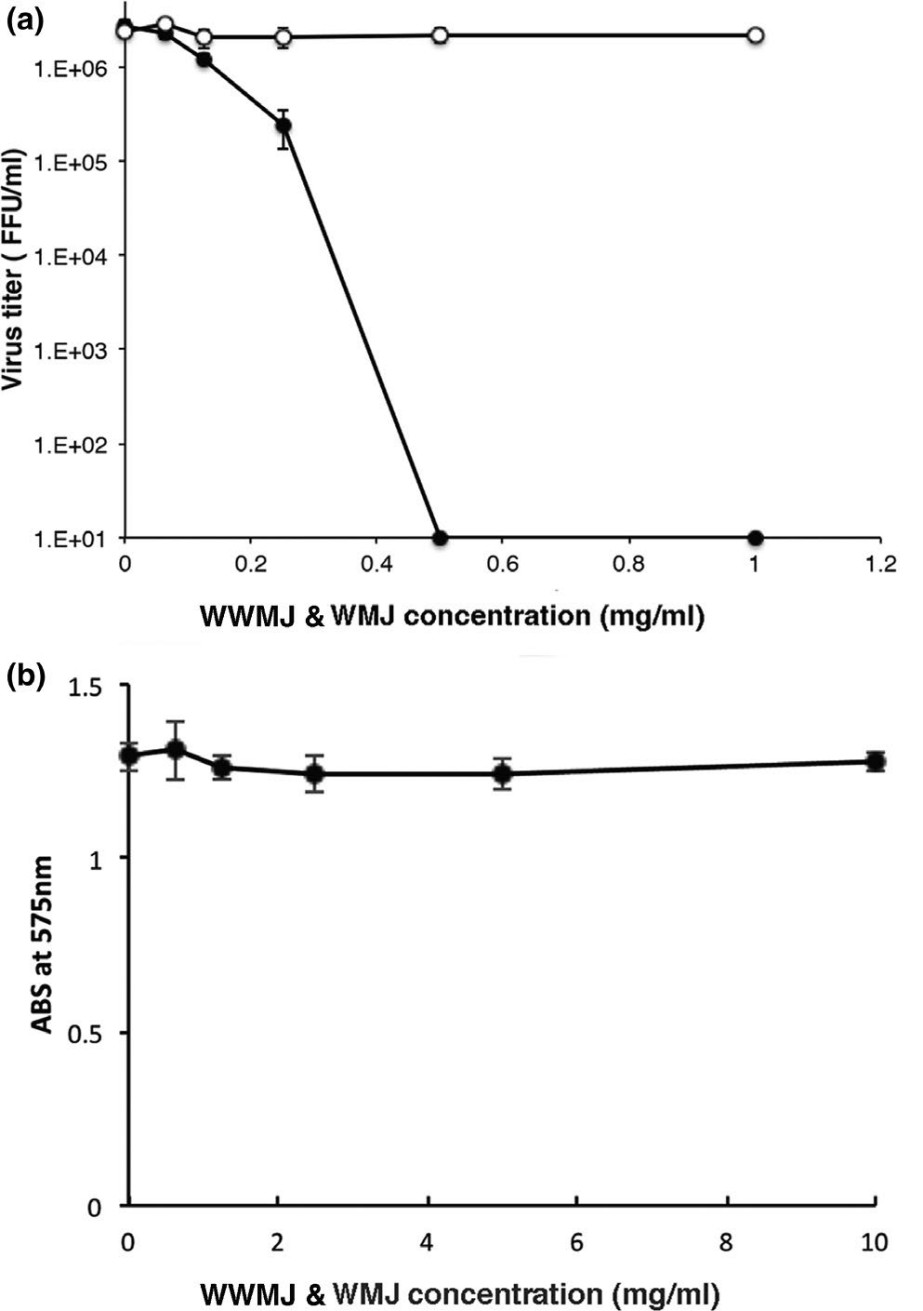
Anti‐influenza virus effects of WWMJ and WMJ. (a) Focus‐forming assay showing viral yields of MDCK cells infected with influenza virus A/PR/8/34 at a MOI of 0.001 24 hr after infection. WWMJ, filled circles; WMJ, open circles. (b) Cell viability of the influenza virus‐infected MDCK cells treated with WWMJ at the indicated concentrations. Error bars show the *SD* (*n* = 3). Data are representative of three independent experiments

The viral growth in infected MDCK cells treated with WWMJ and WMJ (squeezed from a commercially available WM) was compared. WWMJ inhibited the proliferation of the influenza virus within cells in a concentration‐dependent manner, but WMJ did not cause viral inhibition (Figure 1a). The IC_50_ of WWMJ was 0.10 mg/ml. The MTT test revealed that WWMJ was not cytotoxic to MDCK cells (Figure 1b). Notably, 1 mg/ml WWMJ reduced the virus by 80%–90% as compared to control cells lacking WWMJ. The IC_50s_ of winter squash and zucchini were 1.07 and 0.74 mg/ml, respectively. WWMJ had the highest viral growth inhibition activity among plants of Cucurbitaceae that we assayed.

### 3.2 Antiviral effects of WWMJ against various type A and B influenza viruses

The antiviral effects of WWMJ on various influenza strains were then investigated. Table 1 shows the IC_50_ values of WWMJ for various influenza viral strains. WWMJ inhibited the growth of type A and B influenza viruses. IC_50_ values were found to be 0.10–0.20 mg/ml for H1N1, 0.71–0.87 mg/ml for H3N2, and 0.41–0.88 mg/ml for type B viruses. There was no significant difference in the IC_50_ values between the H1N1 viruses (*p* > .05). WWMJ also showed similar inhibitory activities against the oseltamivir‐resistant (A/Osaka/2024/2009 and A/Osaka/71/2011) and ‐sensitive strains (A/PR/8/34, A/New Caledonia/20/99, A/Beijing/262/95, A/Suita/6/2007, and A/Suita/114/2011), for which the IC_50_ values were found to be similar (Table 1).

### 3.3 Influenza growth stage inhibited by WWMJ

**FIGURE 2:**
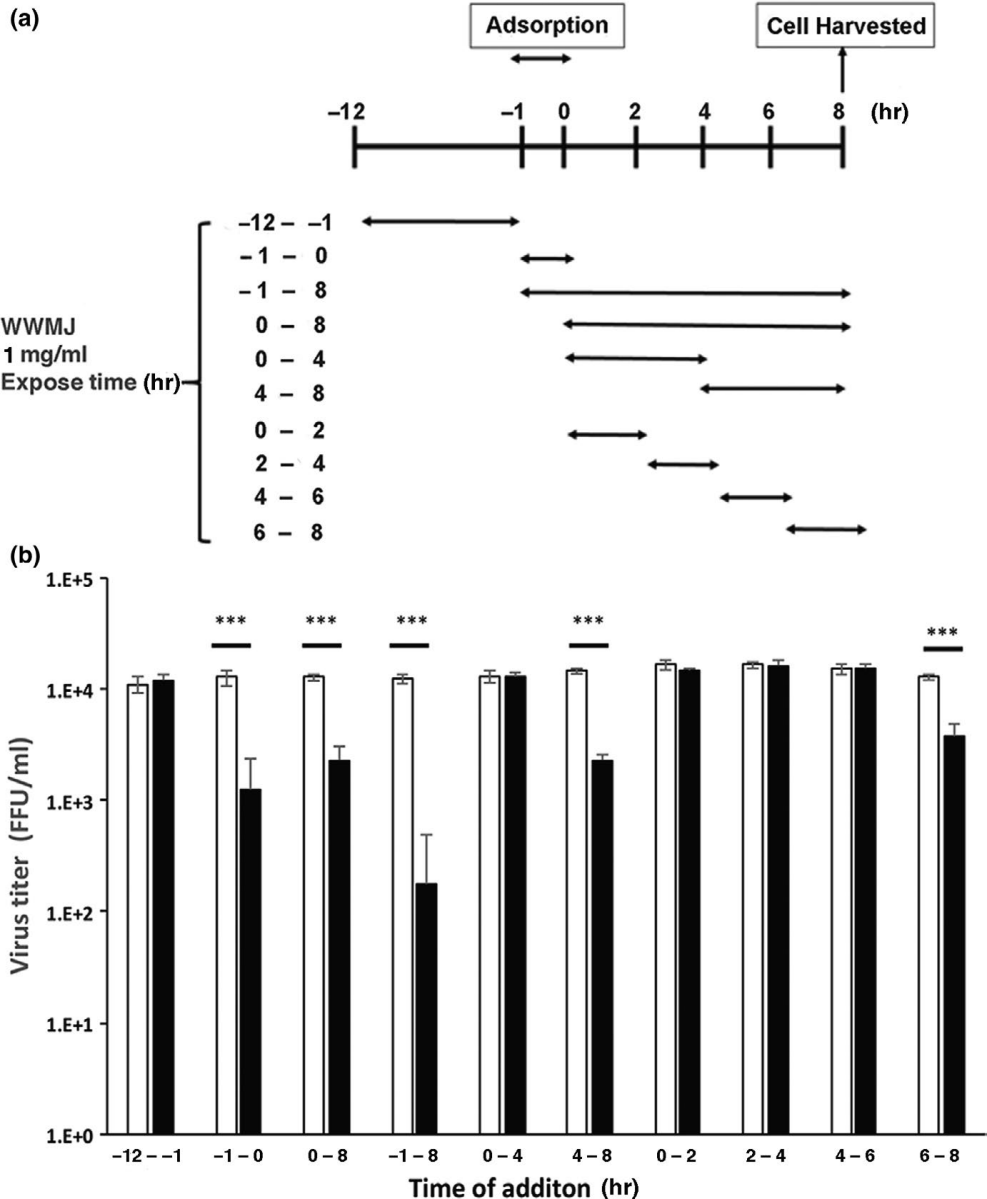
Effect of WWMJ on viral growth stage and virus titer. Time points and exposure periods of addition of MMWJ to MDCK cells infected with influenza virus A/PR/8/34. (a) Time‐of‐addition assay schedule. (b) FFRA‐assay results indicated by open columns representing viral yields of control cells and closed columns representing viral yields of cells treated with WWMJ. Data are representative of three independent experiments. *p* < .001

The stage at which viral growth is inhibited by WWMJ was determined by performing a time‐of‐addition assay. The time points at which WWMJ was added to the incubation mixture are shown in Figure 2a. One cycle of viral (A/PR/8/34) proliferation within a cell takes 8 hr as reported previously (Nagai, 2018). Based on this information, Figure 2b shows the stages of viral multiplication that were inhibited by WWMJ. The exposure period during viral replication was then divided into 2‐hr intervals; the results obtained showed that WWMJ inhibited two different steps in the viral infection process. The first step was the adsorption of the virus onto the cells (‐1–0 hr of viral infection) and the second was in the late stage of viral replication (4–8 hr after viral infection), especially the period associated with viral assembly (6–8 hr after viral infection). Since WWMJ inhibited the adsorption of the virus onto the cells (Figure 2b), the inhibitory mechanism at this step was then elucidated. Type I high‐mannose‐specific antiviral algal lectins have been reported to bind with high affinity to the viral envelope HA (Mu, 2017). The presence of similar lectins in WWMJ was determined by performing a viral yield assay in MDCK cells using heat‐treated WWMJ, which showed that the virus‐inhibiting activity of WWMJ was not affected even after heat treatment.

### 3.4 WWMJ inhibits the cellular entry of influenza virus

**FIGURE 3:**
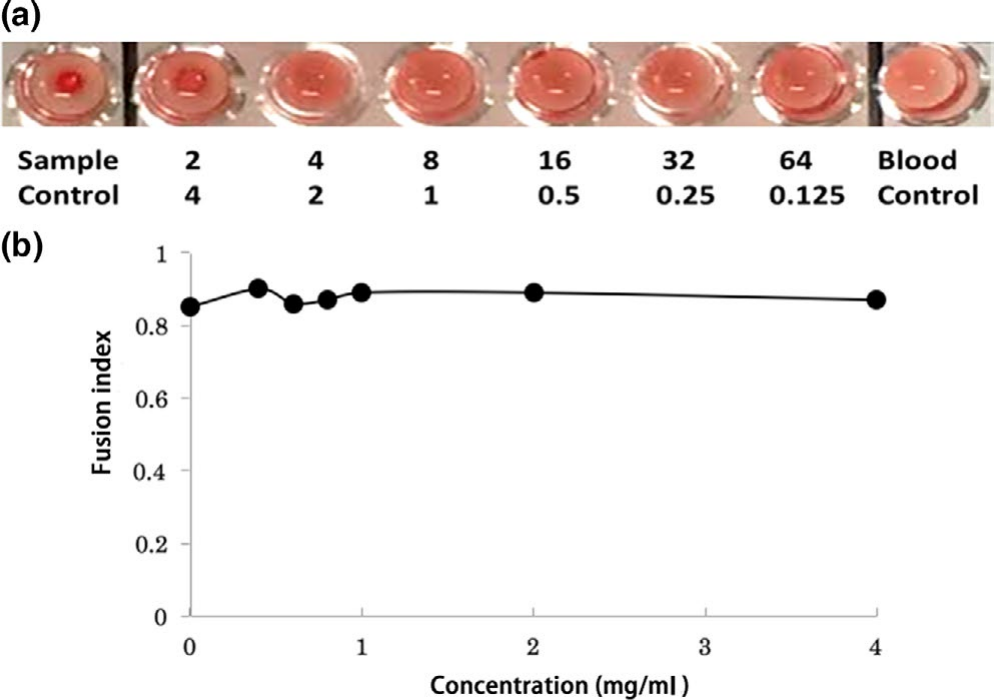
Effects of WWMJ on viral adsorption. (a) Hemagglutination inhibition (HI) assay. “Sample control” represents red blood cells incubated with WWMJ and “Blood control” represents red blood cells infected with virus. Top numbers represent the HI titers and bottom numbers represent the WWMJ concentrations (mg/ml). (b) Fusion inhibition assay. The fusion index = 1 − (number of cells/number of nuclei). Data are representative of two independent experiments

**FIGURE 4:**
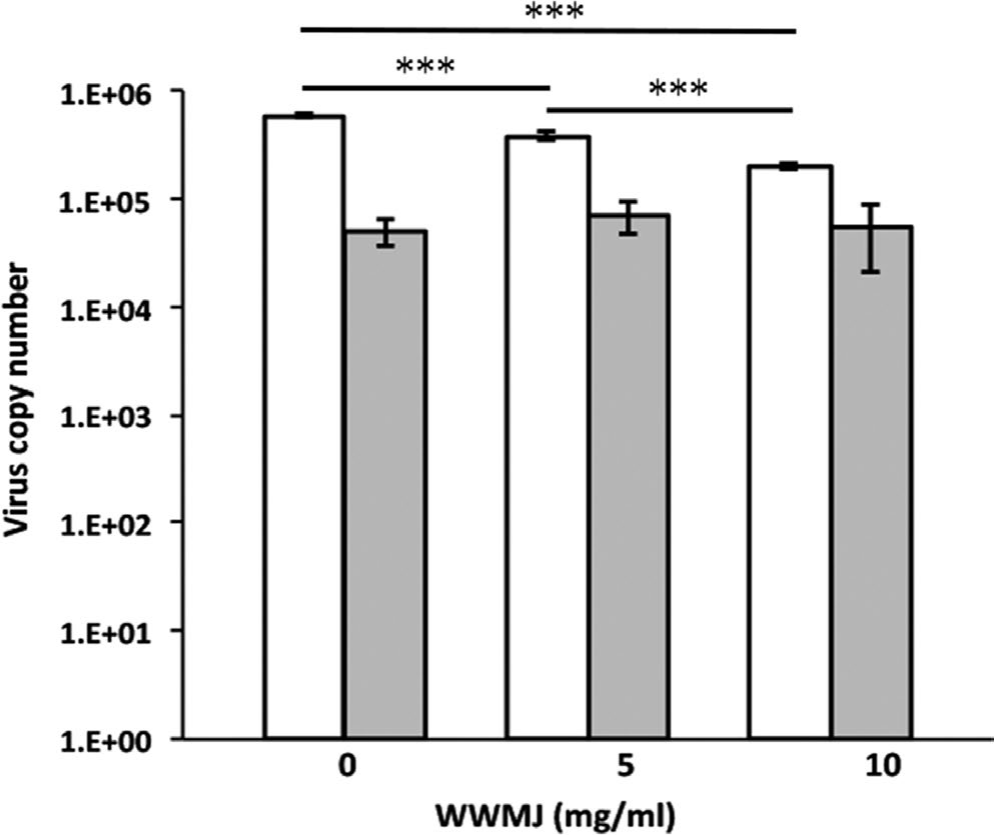
Effect of WWMJ on viral binding to cells. White columns indicate the viral adsorption onto cells at 37°C at three different concentrations of WWMJ. Gray columns indicate the same at 4°C. Error bars indicate *SD* (*n* = 3). Data are representative of two independent experiments. (*p* < .001)

Wild watermelon juice inhibits viral adsorption onto cells as shown in Figure 2b, which was confirmed by the HI test. This test showed that 4 mg/ml WWMJ reacted weakly with the A/PR/8/34 virus (Figure 3a). However, the adsorption inhibition assay showed that WWMJ significantly inhibited the adsorption of influenza virus onto MDCK cells at 37°C, in a WWMJ‐concentration‐dependent manner (Figure 4). The time‐of‐addition assay showed that WWMJ (1 mg/ml) reduced viral adsorption with an inhibition ratio of 0.903 (Figure 2b), and although no inhibition of viral binding occurred in the presence of WWMJ at 4°C, 10 mg/ml WWMJ reduced the viral binding with the cells at 37°C, with an inhibition ratio of 0.648 (Figure 4). The inhibition ratio was defined as (A − B)/A × 100, where A represents the amount of virus in the mock‐treated cells and B represents the amount of virus in the WWMJ‐treated cells. Viral adsorption was analyzed by the time‐of‐addition assay at 37°C since this step could comprise both the attachment of the virus to the cells and virus internalization, such as by endocytosis. However, the viral adsorption assay showed that the amount of bound virus at 37°C was 10 times higher than that at 4°C. WWMJ did not suppress cell–cell fusion using the A/PR/8/34 virus and CV‐1 cell at any of the concentrations tested (up to 4 mg/ml), and the fusion index was found to be more than 0.85 at every concentration (Figure 3b). These results suggested that WWMJ inhibited viral entry by endocytosis but did not inhibit viral fusion ability.

### 3.5 Effects of WWMJ treatment on influenza virus‐infected mice

**FIGURE 5:**
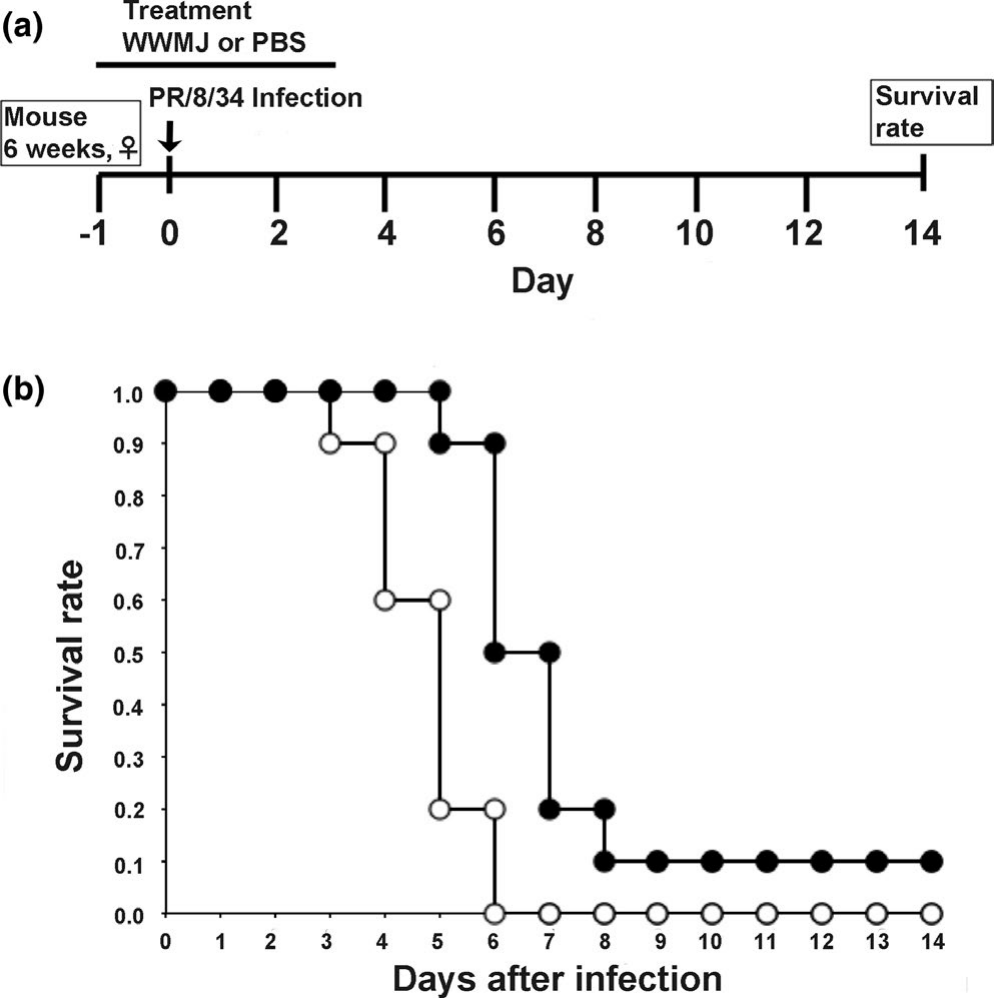
In vivo effect of WWMJ on infected mice. (a) In vivo experiment schedule. (b) Survival rates of BALB/c mice (6 weeks old) infected with mouse‐adapted influenza virus strain A/PR/8/34 at 100 FFU/mouse by nasal mucosal administration for a 14‐day period are shown. Control group treated with PBS, open circles (*n* = 10 mice); WWMJ group, filled circles (*n* = 10). Log‐rank test showed that this difference in survival was significant (*p* < .01)

The therapeutic effects of These WWMJ in vivo were investigated using a mouse model of influenza viral infection. The experimental schedule is shown in Figure 5a. Mice were treated with 20 mg/kg/day WWMJ (test group) or PBS (control group), and their survival rates were evaluated. The body weights of the WWMJ‐treated and control mice did not show any significant difference (unpublished data). As shown in Figure 5b, the nasal administration of WWMJ slightly improved the survival rate of the infected mice monitored for 14‐day postinoculation. WWMJ‐treated mice were found to die 6‐day postinoculation, as compared to death 4‐day postinoculation observed with control mice. The survival rates of the WWMJ‐treated mice were 0.90 and 0.50 at 6‐ and 7‐day postinoculation, respectively, as compared to 0.20 and 0, respectively, in the control mice. Thus, WWMJ treatment increased the lifespans of influenza virus‐infected mice by at least 2 to 3 days. Kaplan–Meier method and log‐rank test showed that this difference in survival was significant (*p* < .01).

## 4 DISCUSSION

Influenza is an acute respiratory disease that affects many people throughout the world. Several anti‐influenza drugs are used to treat patients infected with various influenza viruses. However, viral strains resistant to these drugs have been reported (Barr, 2007; Seibert, 2012).

The anti‐influenza efficacy of WWMJ in A/PR/8/34‐infected MDCK cells was evaluated in this study. WWMJ was found to be the strongest inhibitor of the growth of influenza virus in MDCK cells among all the screened plant extracts of Cucurbitaceae. WWMJ inhibited the proliferation of the influenza virus within the cells in a concentration‐dependent manner, while WMJ did not inhibit the virus.

Viral infections are inhibited by carbohydrates, such as the marine‐microalga‐derived sulfated polysaccharide p‐KG03 (Kim, 2012). The sugar contents of WWMJ and WMJ were determined and found to be 2°Bx and 8°Bx, respectively, on the Brix scale; however, neither contained sulfated polysaccharides (unpublished data). WMJ was found to have four times the sugar content of WWMJ, which indicates that the antiviral effect of WWMJ is not attributable to the influence of sugar. Figure 3 shows that WWMJ did not inhibit the recognition of sialic acid on the cell membrane by HA protein. As a conclusion, Figure 4 suggests that WWMJ inhibits energy‐dependent entry of viruses, in other words, endocytosis.

The finding that WWMJ has a strong anti‐influenza activity, whereas the commercially available WMJ has little anti‐influenza activity, implies that watermelons have lost their effective antiviral components over the course of breeding to suit our taste. Consistent with this idea, it has been reported that wild plants have anti‐influenza properties (Haasbach, 2014). Therefore, wild plants such as WWM and various other spices may have strong antiviral activities against influenza viruses.

In this study, further experiments were carried out to clarify the mechanism and stage of the anti‐influenza activity of WWMJ. Figure 2 shows that WWMJ inhibits adsorption (or entry) and late phase of influenza virus propagation in infected cells. The results of Figures 2 and 4 suggested that clathrin‐dependent and/or ‐independent endocytosis (Fujioka, 2018) might be inhibited by WWMJ. Inhibition of the late phase of influenza virus propagation might involve inhibition of viral assembly, and the mechanism might be similar to the inhibition mechanism of daidzein (Horio, 2020). The assessment of the effect of WWMJ on various virus strains showed that WWMJ inhibited the growth of type A and B influenza viruses. However, no significant difference in IC_50_ values was found between H1N1 viruses (*p* > .05). Although IC_50_ values of H1N1 viruses were significantly 2‐ to 4‐times lower than those of H3N2 and type B viruses (*p* < .05), no significant difference was found between the IC_50_ values for H3N2 and type B viruses (*p* > .05), indicating that WWMJ possesses stronger antiviral activity than adlay tea (Nagai, 2018). Therefore, the viral replication‐inhibition activity of WWMJ does not show virus‐type specificity, unlike that of amantadine (Barr, 2007).

Wild watermelon juice also showed similar inhibitory activities against the oseltamivir‐resistant (A/Osaka/2024/2009 and A/Osaka/71/2011) and ‐sensitive strains (A/PR/8/34, A/New Caledonia/20/99, A/Beijing/262/95, A/Suita/6/2007, and A/Suita/114/2011), which had similar IC_50_ values, suggesting that the effective component(s) in WWMJ that inhibit viral growth act differently from the inhibitory components of oseltamivir. Moreover, since WWMJ shows similar effectiveness in inhibiting both type A and B influenza virus growth (Table 1), WWMJ might have utility as a new preventive and/or therapeutic substance, which can combat the problem of emergence of mutated drug‐resistant strains.

The time‐of‐addition assay showed that WWMJ inhibited viral adsorption and late viral replication, indicating that it may contain two or more components that act as the main inhibitors of viral adsorption and assembly. The findings of this study indicate that WWMJ might have potential utility as a novel alternate treatment for influenza virus, although further biochemical and molecular biological studies are required to further understand its active components and antiviral mechanisms.

Wild watermelon juice was found to reduce the viral titer in vitro and improve the survival of influenza virus‐infected mice in vivo. The results obtained in this study therefore demonstrate an alternate mode of management and treatment of influenza infections that may be useful for combating the virus. Thus, this study might have proved the candidature of WWMJ as a potentially novel and alternate mode of treatment for influenza infection. Since WWMJ has been indicated to comprise multiple antiviral components, this alternate mode of treatment can ensure the prevention of new resistant viral strains. This alternate mode of treatment promises to be nonspecific to viral strains, thereby resulting in a therapeutic option for infections caused by newly emerging mutated influenza virus strains as well.

## STUDIES INVOLVING ANIMAL OR HUMAN SUBJECTS

This study was performed with the approval of the Animal Experiment Committee of Hyogo College of Medicine, Nishinomiya, Japan (approval number: 15–007) and complied with the ordinance of the Regulation for Enforcement of the Act on Welfare and Management of Animals relating to the care and management of experimental animals.
